# Hematopoietic stem cell transplantation for multiple sclerosis: is it a clinical reality?

**DOI:** 10.1186/s13287-015-0272-1

**Published:** 2016-01-16

**Authors:** Maha M. Bakhuraysah, Christopher Siatskas, Steven Petratos

**Affiliations:** Department of Medicine, Central Clinical School, Monash University, Prahran, VIC 3004 Australia

## Abstract

Hematopoietic stem cell transplantation (HSCT) is a treatment paradigm that has long been utilized for cancers of the blood and bone marrow but has gained some traction as a treatment paradigm for multiple sclerosis (MS). Success in the treatment of patients with this approach has been reported primarily when strict inclusion criteria are imposed that have eventuated a more precise understanding of MS pathophysiology, thereby governing trial design. Moreover, enhancing the yield and purity of hematopoietic stem cells during isolation along with the utility of appropriate conditioning agents has provided a clearer foundation for clinical translation studies. To support this approach, preclinical data derived from animal models of MS, experimental autoimmune encephalomyelitis, have provided clear identification of multipotent stem cells that can reconstitute the immune system to override the autoimmune attack of the central nervous system. In this review, we will discuss the rationale of HSCT to treat MS by providing the benefits and complications of the clinically relevant protocols, the varying graft types, and conditioning regimens. However, we emphasize that future trials based on HSCT should be focused on specific therapeutic strategies to target and limit ongoing neurodegeneration and demyelination in progressive MS, in the hope that such treatment may serve a greater catchment of patient cohorts with potentially enhanced efficiency and lower toxicity. Despite these future ambitions, a proposed international multicenter, randomized clinical trial of HSCT should be governed by the best standard care of treatment, whereby MS patients are selected upon strict clinical course criteria and long-term follow-up studies of patients from international registries are imposed to advocate HSCT as a therapeutic option in the management of MS.

## Background

Multiple sclerosis (MS) has been defined as an autoimmune disease of the central nervous system (CNS). Although the etiology of MS has not been clearly elucidated, it is generally agreed that autoreactive T cells, activated by either self-reactive or cross-reactive antigens, migrate through the blood–brain barrier (BBB) and trigger an inflammatory cascade that ultimately leads to demyelination and progressive neurodegeneration of the CNS [1]. Pathologically, the brain tissue of autopsy patients exhibits inflammatory infiltrates with the degeneration of myelin, reactive gliosis, and axonal degeneration [2, 3]. Neurological disability is manifest in a number of symptoms, including blurred vision and diplopia, sensory disturbances (e.g., paresthesia and dysesthesia), heat intolerance, hemiparesis or paraparesis, vertigo and dizziness, lack of coordination, limb spasticity, bowel and bladder incontinence, cognitive impairment, and memory loss.

As with most autoimmune disorders, MS predominantly affects young females between 20 and 40 years of age with the prevalence being 80–120/100,000 population with a lifetime risk of 1 in 400 [4, 5]. MS is classified into four main subtypes: relapsing remitting (RR), secondary progressive (SP), primary progressive (PP), and progressive relapsing (PR) [2]. Over 80 % of patients with MS begin with a RR course characterized by relapses that result from inflammation, followed by incomplete or complete remission. After 5–15 years from its onset, 50 % of patients enter SP-MS, where pre-existing neurological deficits gradually worsen from the onset with subsequent superimposed relapses. The latter is characterized by axonal degeneration and loss, leading to gliosis and brain atrophy. MS follows the PP phase in 15 % of individuals, in which disability accumulates faster than in the early RR course. PR-MS is the least frequent form of MS and is characterized by a steady neurological decline with superimposed attacks experienced by the patient [3, 6].

Currently there is no cure for MS, but a number of therapeutic agents are used to treat specific symptoms and sequelae of the disease, with most designed to prevent the progression of disability by targeting immune activation and inflammation [3]. Conventionally, MS can be treated by chemotherapeutic agents for chronic immunosuppression, corticosteroids for the management of acute inflammatory relapses, and immunotherapeutic interventions for immunomodulation, using drugs such as natalizumab, interferon beta, glatiramer acetate, dimethyl fumarate, alemtuzumab, and fingolimod [3]. These treatments are used to diminish the patient’s relapses both in frequency (e.g., glatiramer, interferon beta, and more recent types of monoclonal antibodies given regularly) and in severity (e.g., corticosteroids taken acutely) [7]. Among the most promising strategies used in regenerative medicine, hematopoietic stem cell transplantation (HSCT) prevails as an excellent but controversial therapeutic regime to limit the deleterious pathology following an autoimmune attack. It has been posited that HSCT may in fact be useful for improving the neurological function of MS patients by the replacement of autoreactive cells with healthy cells, potentially removing the patient’s genetic susceptibility to develop MS [8].

## Hematopoietic stem cell transplantation

**Fig. 1:**
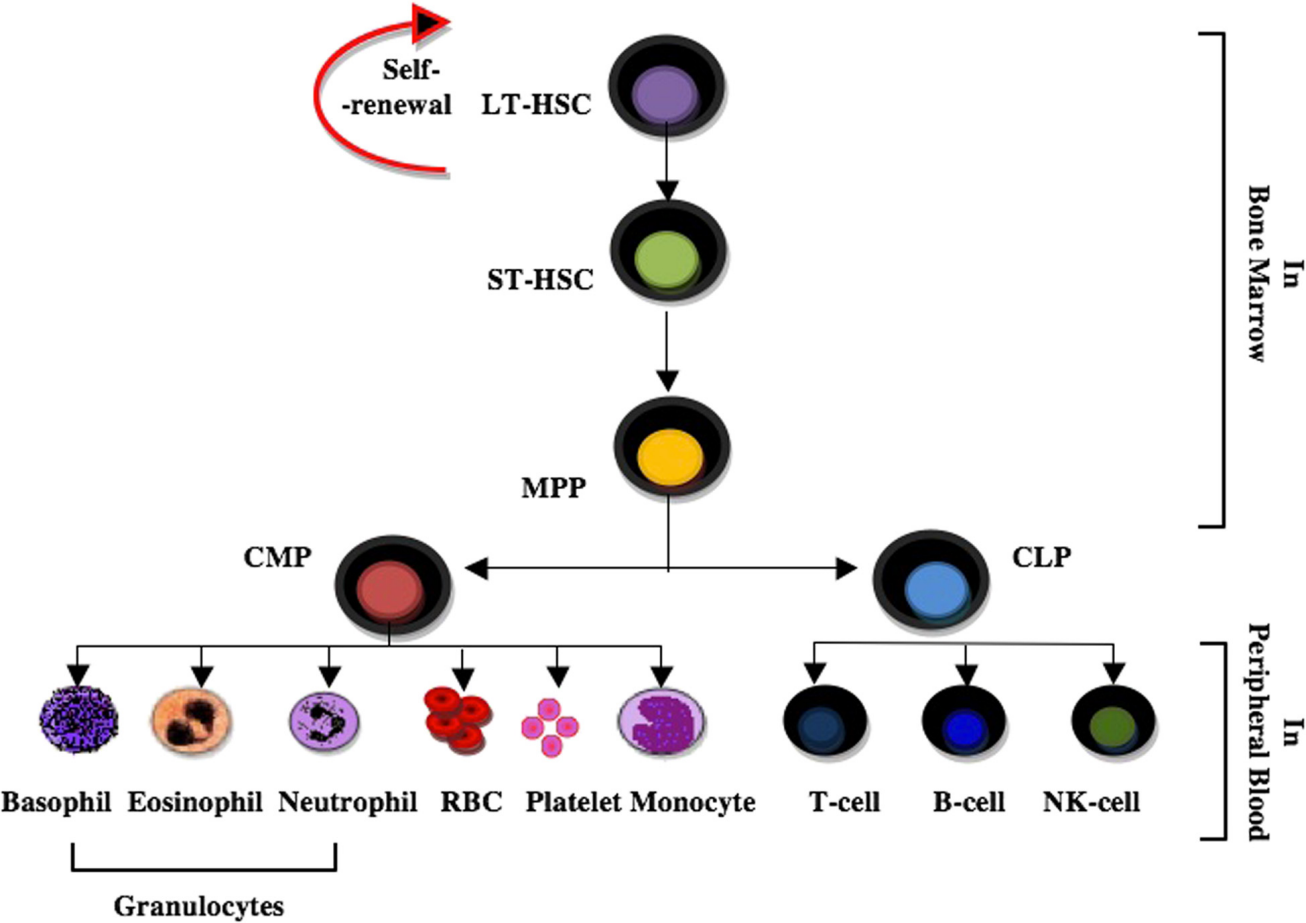
Hematopoietic hierarchy model. Hematopoietic stem cells (*HSCs*) are divided into long-term (*LT*)-HSC and short-term (*ST*)-HSC types. A LT-HSC with long-term self-renewal activity is converted into a ST-HSC and then HSCs give rise to a multipotent progenitor (*MPP*). A MPP commits in bone marrow to become either common myeloid progenitor (*CMP*) or common lymphoid progenitor (*CLP*). The CMP and CLP give rise to mature blood cells in peripheral blood, such as granulocytes, red blood cells (*RBC*), platelets, monocytes, T cells, B cells, and natural killer (*NK*) cells [7]

HSCT has been harnessed for more than 40 years in the clinic as an effective therapeutic approach. In 1995, the first transplantation of hematopoietic stem cells (HSCs) was suggested as a treatment for MS after hypothesizing that an immune-mediated attack on myelin causes pathologic events in MS [9]. Two years later in the United States, HSCT was performed in 15 MS patients with a progressive form [10]. HSCs capable of self-renewal when effectively transplanted and engrafted in the human can differentiate into all of the cells found in the hematopoietic system. They are divided into two different types: long-term (LT) and short-term (ST) subtypes (Fig. 1). LT-HSCs have the ability to self-renew and provide all hematopoietic lineages during the life of an individual. ST-HSCs, as the name suggests, are incapable of long-term self-renewal under normal conditions, but they do provide the ability to reconstitute hematopoiesis of certain lineages over a finite period [11].

## Isolation of HSCs

HSCs represent rare cell populations that exist in the bone marrow (BM) and constitute approximately 0.01 % of total nucleated cells [12]. They can afford the complete restoration of all blood-cell lineages after BM ablation in vivo and the improvement of the MS patients’ immunity. Based on this definition, several xenogenic and congenic assays have been established to quantitate and detect human and mouse HSCs [13].

HSC populations isolated from their stem cell niche differ morphologically. They can therefore be separated using several methods based on their physical properties, on their physiological properties, or using specific cell surface markers [12]. The study of HSCs has been expedited in the last 20 years by the development of various isolation techniques such as flow cytometry and the availability of newly developed monoclonal antibodies more specific to HSCs, with the most successful approaches varying in selectivity, choice of separation parameters, and capacity [13]. These methods are classified into nonantibody-based and antibody-based HSC selection methods.

### HSC selection methods without the need of antibodies

The most common technique for separating HSCs, based on differences in their cell size and density, is density gradient centrifugation [13, 14]. HSC suspensions are centrifuged through a density medium such as Percell (GE Healthcare life Sciences, Uppsala, Sweden) or a mixture of Ficoll (GE Healthcare life Sciences) and Hypaque (GE Healthcare life Sciences), resulting in the separation of denser mature cells such as granulocytes and erythrocytes. However, this method results in loss of HSCs due to overlapping densities of lymphocytes and stem cells. Cytotoxic drugs, such as hydroxyurea and 5-fluorouracil (5-FU), can be used to selectively kill mature cells which are undergoing cell division and this approach has been used to enrich mouse HSCs before other selection techniques. Hydroxyurea inhibits the activity of ribonucleotide reductase, whereas 5-FU inhibits thymidylate synthase, the end result being decreased production of deoxyribonucleotides, which are used in replication. Since most HSCs are quiescent under steady-state conditions, they are resistant to cytotoxic drugs. Alkylating agents such as phosphamide can interfere with DNA replication and can also be used to eliminate dividing cells. In addition, HSCs express aldehyde dehydrogenase (ALDH), which is an intracellular enzyme that confers resistance against phosphamides [13]. It has been demonstrated that fraction 25, lineage-depleted, ALDH bright cells are stable for long-term reconstitution of lymphohematopoietic cells at 10 cells per animal. These techniques can be utilized to distinguish primitive HSCs from multiprogenitors demonstrated through colony-forming spleen assays (CFU-S) [14]. Furthermore, HSCs are enriched by the activity of ALDH; the expression of this enzyme overlaps with the expression of CD34 in the BM cells of adult humans, demonstrating that the activity of this enzyme is a marker for primitive HSCs as well as lineage-committed progenitor cells [13].

### Antibody-based HSC selection methods

The antibody-dependent methods for the isolation of HSCs rely on the availability of monoclonal antibodies directed against specific cell surface markers followed by isolation using either fluorescence-activated cell sorting (FACS) or magnetic-activated separation (MACS) [13]. Utilizing flow cytometry, fluorescently tagged antibodies are used to identify surface proteins, which are expressed at specific stages of development, permitting distinctions among phenotypically homogeneous cell populations. Using MACS, antibodies directed against a surface antigen of interest are coated with magnetic nanoparticles. The cells are separated by placing the cell suspension into a magnetic field following antibody incubation. Both FACS and MACS use positive and negative selection. These techniques are typically applied to provide a sufficient yield and purity of HSCs for clinical transplantation purposes by eliminating the vast majority of mature cells. Furthermore, these techniques can be adapted for either positive or negative selection of HSCs [13].

## Cell surface markers of human and mice HSCs

Although there are several shared biological similarities between human and mouse HSCs, there are differences in the purification strategies implemented for human HSCs as opposed to the experimental isolation of mouse HSCs when utilized for therapeutic applications. Although the human cord blood or adult BM side population showed very low HSC activity in vitro, the mouse BM side population represents significant enrichment for hematopoietic activity. Another disparity is that positive selection through the enrichment of human CD34^+^CD38^–^ cells has been readily utilized for clinical purposes enriching the populations of progenitors and HSCs [13]. However, it has been shown that only low levels of CD34 are expressed on mouse LT-HSCs [12]. An outline of readily utilized markers for both human and mouse HSCs is presented in Table 1. The main HSC markers that distinguish mouse and human cells are presented in Table 2.

**Table 1:** Surface profile of HSCs in mouse and human [7]

CD marker	Synonym	Main expression	Function
CD3	T3, leu4	T cell	Mediated T-cell signal transduction and used in Lin cocktail
CD4	T4, leu3	MHC-II, T cell, macrophage/monocytes, dendritic cells	Initiate early phase of T-cell activation and used in Lin cocktail
CD8	T8	MHC-I, T-cell subsets	T-cell-mediated killing, and used in Lin cocktail
CD11b	CR3, MAC 1	Macrophage/monocytes, dendritic cells, granulocytes, NK cells	Phagocytosis, adhesion interaction of macrophage/monocytes, granulocytes, and used in Lin cocktail
CD11c	CR4	Macrophage/monocytes, granulocytes, NK cells	Similar to CD11b, cell–cell interaction during inflammatory response, and used in Lin cocktail
CD34	Gp105/120, Mucosialin	Precursor of hematopoietic cells, endothelial cells	Cell adhesion
CD38	T10	Lymphoid cells, macrophage/monocytes	Cell adhesion and transduction
CD45R	B220, Ly-5	T cells and mostly B cells	T-cell and B-cell antigen receptor-mediated signaling, and used in Lin cocktail
CD59	MIRL	T cells, NK cells, granulocytes, erythroid, macrophage/monocytes	Complement cascade regulation
CD117	c-Kit	HSCs/progenitor cells and mast cells	Survival of mast cells, activation, proliferation, and chemotaxis
CD161	NK1.1	NK cells	NK cell-mediated cytotoxicity, proliferation, and used in Lin cocktail
SCA-1	Ly6A/E	HSCs, HPCs, some lymphoid and myeloid cells	Mice HSCs are positive
Gr1	Ly-6G	Monocytes and granulocytes	Used in Lin cocktail
Ter119	Ly76	Erythroid cells	Used in Lin cocktail

*CD* cluster of differentiation, *c-Kit* tyrosine-protein kinase receptor, *CR* complement receptor, *HPC* hematopoietic progenitor cell, *HSC* hematopoietic stem cell, *leu* leucine, *Lin* lineage markers, *Ly* lymphocyte activation protein, *MAC1* macrophage 1 antigen, *MHC* major histocompatibility complex, *MIRL* membrane inhibitor of reactive lysis, *NK* natural killer, *SCA-1* stem cell antigen-1

**Table 2:** Main markers used to discriminate mouse and human HSCs [10]

	Cell surface markers
Mouse	lin^–^, CD34^–/low^, CD38^+^, Sca-1^+^, c-Kit^+^, Thy-1, FGFR, CD201, CD105
Human	lin^–^, CD34^+^, CD38^–/low^, CD133, c-Kit^–/low^, Thy-1^+^, CDCPI, VEGFR1

*CD* cluster of differentiation, *CDCPI* cubdomain-containing protein, *c-Kit* tyrosine-protein kinase receptor, *FGFR* fibroblast growth factor receptor, *HSC* hematopoietic stem cell, *lin* lineage markers, *Sca-1* stem cell antigen-1, *Thy-1* thymocytes, *VEGFR1*; vascular endothelial growth factor receptor 1

In mouse HSCs, most of the purification approaches revolve around the positive selection markers, such as stem cell antigen-1 (SCA-1) or the transmembrane tyrosine-protein kinase receptor (c-Kit or CD117) and negative markers for mature HSC lineages (e.g., CD3e, CD4, CD8a, CD45R/B220, CD11b, CD11c, NK1.1, TER119, and Gr-1). Removal of mature cells that express lineage (lin) markers using an antibody cocktail leads to the enrichment of blast cells, HSCs, and progenitor cells from BM and blood [13]. Therefore, about 10 % of Lin^–^, Sca-1^+^, and c-Kit^+^ (LSK) cells are bonafide LT-HSCs, which are self-renewing cells and can grow in culture, along with being able to be transduced ex vivo using lentiviral vectors [15]. However, CD34^+^ cells from mouse BM are expressed on ST-HSCs, which rapidly die ex vivo and then cannot be cultured [16]. Hence LSK cells are those selected for sorting by FACS for long-term culture experiments.

In addition to the identification of cell surface markers, the side population has been used to further enrich BM for mouse or human HSCs based on their capability to efflux the Hoechst nuclear dye via a membrane transport pump (ATP-binding cassette family). The dye is reserved at low levels in these cells in a highly active form when compared directly with other types of BM cells [12, 13]. Rhodamine-123 (Rho) and Hoechst 33342 (Ho) are examples of effluxing specific fluorescent dyes. Rho^–/low^ presents the majority of HSCs from adult mouse and human, while this phenotype of Rho^–/low^ is regulated correspondingly to CD34 in murine HSCs [13]. According to Pearce and Bonnet [17], it may be that there is no long-term reconstitution established by utilizing either human HSCs or the cord blood side population, although there is a significant HSC enrichment when using the mouse stem cell side population, which are primarily CD34^–^, highlighting differences in the transplantation of HSCs in experimental models when compared with those performed in the clinical setting.

## HSCT in a mouse model of MS

HSCT was clinically pursued in MS based on strong data obtained from mouse models of autoimmune encephalomyelitis investigating the outcome of the therapy. A series of experiments conducted in the early 1990s used the classical experimental autoimmune encephalomyelitis (EAE) model, which has been widely used to understand the mechanism of MS disease. It is induced by immunization with the myelin oligodendrocyte glycoprotein (MOG), proteolipid protein (PLP), or myelin basic protein (MBP) [8, 18].

**Fig. 2:**
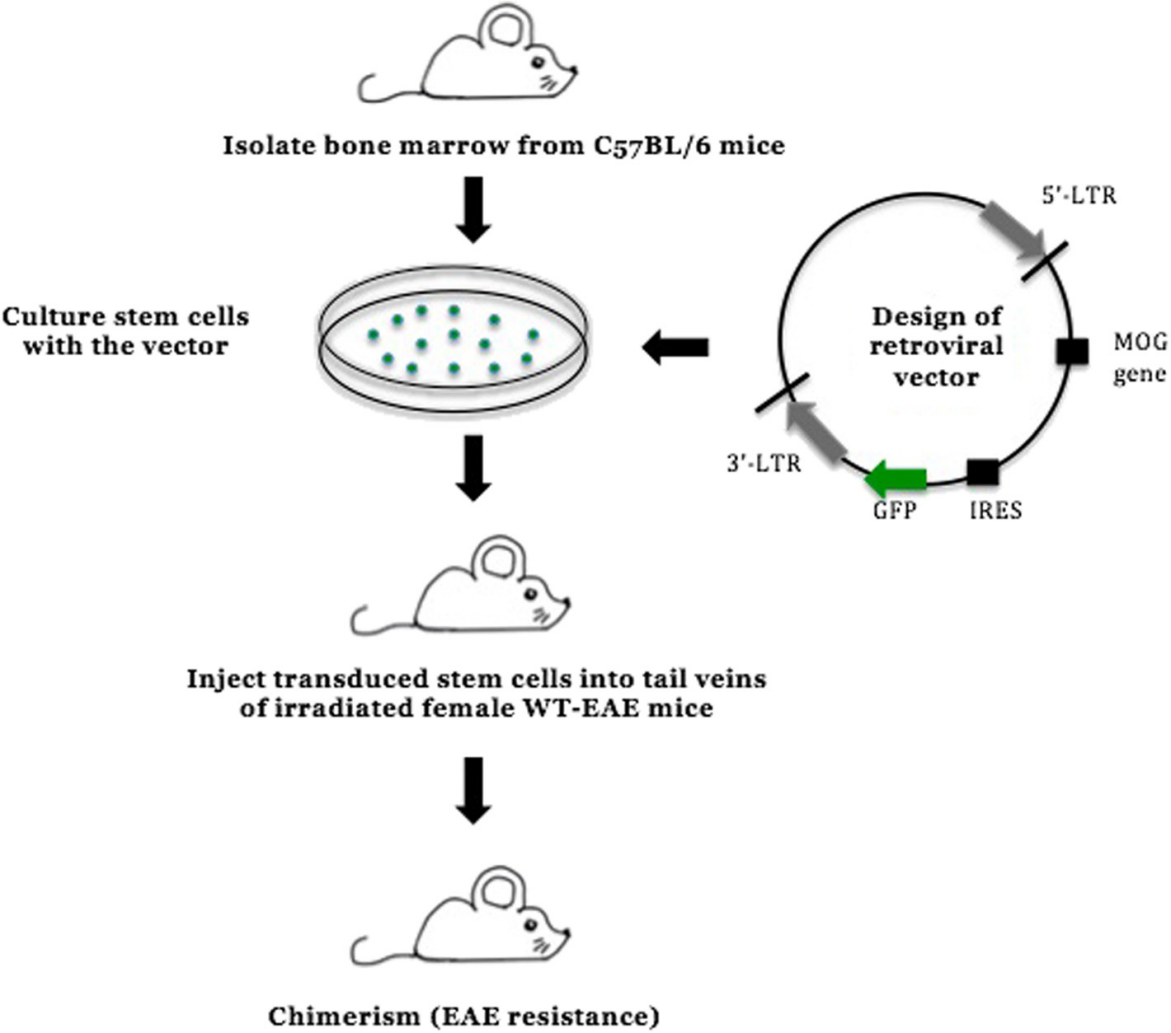
Experimental procedure of bone marrow transplantation in experimental autoimmune encephalomyelitis (*EAE*) mice. Isolated BM cells from donor mice (C57Bl/6) 6–10 weeks old are cultured and ex vivo transduced with a retroviral vector, which encodes the myelin oligodendrocyte glycoprotein (*MOG*) gene and green fluorescent protein (*GFP*) driven by an internal ribosomal entry site (*IRES*) to assist enumeration and tracking of these cells. Transplanted GFP-expressing cells into irradiated mice following their immunization with MOG_35–55_ to induce EAE are assessed in their development or protection of disease, as well as chimerism [20]. *LTR* long terminal repeat, *WT* wild type

EAE can be induced in C57BL/6 mice by immunizing with the MOG_35–55_ peptide emulsified in complete Freund’s adjuvant (CFA), resulting in a progressive paralysis ascending from the tail to the forelimbs. The disease progression in this model can thus be scored accordingly and the immune-mediated pathology within the CNS can be measured accurately. Furthermore, myelin damage and CNS inflammation in this mouse model can be quantified and visualized using histological analysis, in addition to immunological assays to measure autoantibody reactivity and specific T cells which can be used to validate certain immune responses to the different myelin components [19]. Recent research in autoimmune conditions is now also investigating gene therapy as a possible option to correct the defective HSCs present prior to transplantation [20]. The experimental procedure includes the isolation of BM stem cells from donor mice that are transduced with retrovirus encoding a specific antigen after culturing these cells ex vivo. Transduced stem cells are then transferred to conditioned recipient mice, following total body irradiation (TBI), leaving them to engraft and regenerate the hematopoietic system, including dendritic cells, B cells, and T cells, all lineages being identified by flow cytometry (Fig. 2). Using this model, mice were resistant to EAE induction after transferring transduced BM cells with retrovirus encoding MOG [20].

The rationale of autologous hematopoietic stem cell transplantation (auto-HSCT) for MS is based on using chemotherapy to induce immunoablation, with subsequent reconstitution of the impaired immune system through renewed self-tolerant cells [21]. A report in 1996 by van Gelder and van Bekkum [22] explored the use of auto-HSCT in EAE, demonstrating that lymphocytes, which are present in the autologous cells, might lead to the occurrence of relapse after transplantation. An additional report by van Bekkum in 2004 [23] explored the efficacy of auto-HSCT with TBI in Buffalo rats with EAE, and absence of disease relapse was seen in 70 % of cases. The treatment was found to be most effective at the early stages of autoimmune disease, whereas no effect was seen in the later stages. High-dose TBI led to a better response; however, TBI can lead to severe carcinogenic events at a later stage. The use of cyclophosphamide and busulfan also proved less effective than TBI [23].

In addition, the animal model study was conducted to supplement the conventional nonspecific-dose immunosuppression; it involved the administration of high-dose immunosuppressant (myeloablative chemotherapy) followed by HSCT, which effectively reduced morbidity and mortality in this model. The transplant experiments showed that EAE remissions were attained in animals after high-dose TBI. Therefore, the use of high-dose immunosuppressive agents in EAE led to a better response as compared with TBI [24].

## HSCT as a therapeutic option for MS

Over the last 20 years, HSCT was anticipated as a treatment for MS patients by ablating or suppressing the endogenous immune system. It is likely to beneficially affect the inflammatory stage of the disease [25]. In the 1990s, auto-HSCT was suggested for the management of refractory and severe autoimmune disease, including MS. Only a few HSCT trials have implemented the use of donor-derived HSC grafts or HLA-matched allogeneic cell transplants because of the higher rate of mortality and morbidity with graft versus host disease (GVHD) than auto-HSCT. The role of HLA proteins is to direct the response of T cells and they are important in the selection of donors for allo-HSCT [26, 27].

**Fig. 3:**
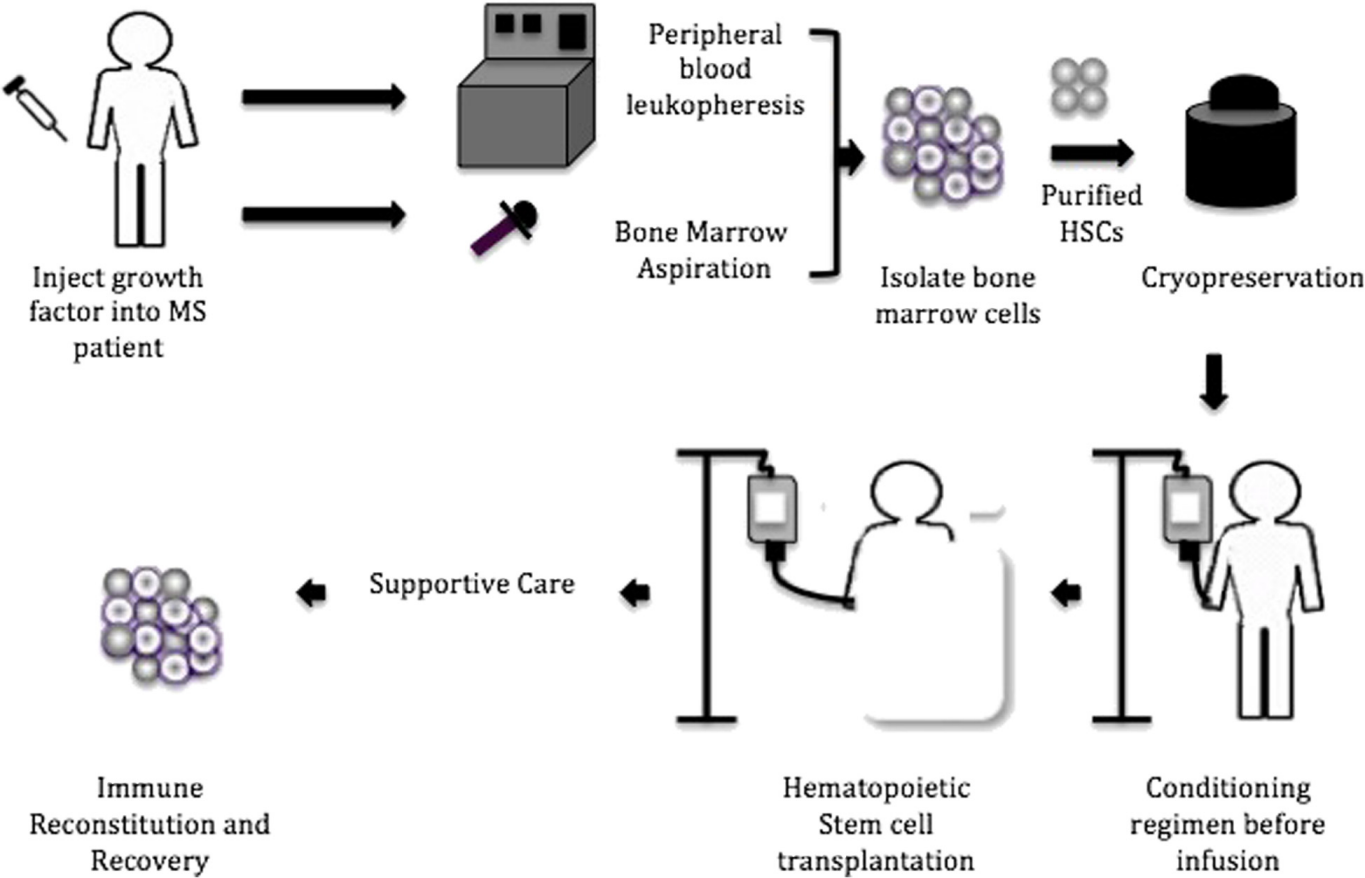
Autologous hematopoietic stem cell (*HSC*) transplantation in multiple sclerosis (*MS*) patients. The technique is initiated after collecting HSCs from the patient’s BM through BM aspiration or peripheral blood leukopheresis achieved under general or regional anesthesia [29]. The collected HSC grafts are cryopreserved in liquid nitrogen until they are required for transplantation [29]. The patient’s immune cells will be destroyed after high-dose chemotherapy along with immune ablative conditioning regimens. The cryopreserved HSCs will be infused into the patient intravenously, and then the reconstitution of the hematopoietic system will occur following 10–14 days after transplantation, with full recovery from chemotherapy occurring between 3 and 6 months [29]

Recently, several MS patients were treated with auto-HSCT after exposure to high doses of immunosuppressive drugs by using different procedures of HSC harvesting and conditioning regimens [26–28]. In a BM graft, around 3–5 % of the cells are HSCs and the graft of peripheral blood HSCs are rich in lymphocytes, granulocytes, and monocytes. The procedure of transplantation is initiated after collecting HSCs from the patient’s BM through several aspirations performed under general or regional anesthesia [29]. Otherwise, peripheral blood stem cells (PBCS) can be mobilized from the BM into blood circulation in large amounts by using chemotherapy and/or a specific cytokine, such as granulocyte colony-stimulating factor (G-CSF), and FLT3 ligand thyroid peroxidase (TPO). In HSCT, this cytokine is injected into the donor prior to harvesting stem cells to maximize HSC collection. During the apheresis process, the donor’s blood is passed through a device whereby CD34^+^ (a cluster of differentiation mixture of HSCs, progenitor cells, and white blood cells) is expunged, and then red blood cells are returned to the donor. By using this method, 5–20 % of the extracted HSCs are suitable for treating patients with MS [29]. These purified HSCs can be cryopreserved until the patients are ready to undergo transplantation, estimated to be between 2 and 6 weeks. In this process, administration of chemotherapy with or without immune-depleting biological agents is vital for destroying the patient’s mature immune lineage cells before infusing the cryopreserved HSCs. Hematopoietic engraftment and recovery from chemotherapy may take between 3 and 6 months [29] (Fig. 3).

Immune reconstitution occurs via two key mechanisms: homeostatic expansion of mature T cells and B cells comprised in the graft; and de novo lymphopoiesis of new engrafted HSCs [30]. In theory, ongoing MS activity after HSCT may be caused by the expansion of autoreactive lymphocytes. Procedures to diminish the immune cell load in the graft may improve the outcome. Therefore, the effect of using immune ablative conditioning regimens to deplete lymphocytes in the graft may be apparent following HSCTs [29]. However, when adopting or developing pre-existing HSCT regimens for novel indications, such as MS, consideration needs to be given to cytogenetic abnormalities, to the patient’s medical condition (e.g., age, status of performance and status of disease), to an existing graft source, and to disease-specific prognostic factors [31].

Muraro et al. [32] showed that using the auto-HSCT myeloablative regimen can reconfigure the immune system in mature MS patients by reconstituting the CD4^+^ T-cell lineage population. In myeloablative conditioning regimens, patients are given chemotherapy with or without TBI before transplantation. The purpose of this process is to eliminate disease in the patient before HSC infusion and suppression of the immune system, and it requires stem cell support to rescue marrow function and avoid aplasia-related death. However, the process is restricted to patients younger than 50 years old due to its toxic effect on non-BM organs (e.g., liver, heart, and lung) [29].

On the other hand, Burt et al. [25] confirmed in their study that the purpose of conditioning regimens in MS is lymphoablative because the rationale of auto-HSCT is to revive an antigen-naive immune system from the patient’s HSCs; thus, myeloablative regimens are lethal to HSCs. In addition, the rationale of nonmyeloablative HSCT in MS is to suppress relapses by intervening prior to the onset of irreversible progressive axonal degeneration, to prevent inflammation, and to reduce toxicity in the older patient population. Such a regimen, being immunosuppressive in nature but without the myeloablative side effects, can be designed to dampen the activity of the immune system by using cyclophosphamide, fludarabine, rabbit anti-thymocyte globulin (ATG), or CAMPATH-1H (anti-lymphocyte antibodies), and/or by using the graft selection of CD34^+^ [25, 33]. This ex vivo cell selection or depletion technology can change the composition of graft cells but may lead to an elevated risk of treatment-related infection for an intense conditioning regime [25]. Furthermore, the intensity of conditioning regimes may exert a toxic effect on the CNS and this neurotoxicity has been associated with radiation and busulfan treatment. Therefore, it may stand to reason that moderating conditioning regimes may provide a less toxic outcome for MS patients when treated through HSCT [25].

Recently, powerful conditioning regimens followed by auto-HSCT have been applied to aggressive MS. An example of these is alemtuzumab, which is a monoclonal antibody that targets the B-cell and/or T-cell compartment. Alemtuzumab contributed to the depletion of circulating lymphocytes that lead to the control of the autoimmune response in MS patients after transplantation, as well as preventing the development of GVHD by diminishing cytotoxic effector cells [21]. However, it has been noted that using this drug may lead to the induction of secondary autoimmune sequelae [34]. Around 10 % of patients undergoing HSCT for autoimmune illness have been observed to develop a secondary autoimmune disease unrelated to their induction for auto-HSCT within the first 2 years after HSCT, although the primary autoimmune illness may have been suppressed following the HSC graft [35]. Secondary autoimmune disease was found to be less common in patients who undertook HSCT for MS but more frequent in patients who undertook HSCT for systemic lupus erythematosus [34]. Furthermore, immune thrombocytopenic purpura (immune cytopenia) may occur many years after HSCT. This may occur as a result of the lymphocyte-depleting antibodies administered during the conditioning of the HSC graft [36]. Secondary autoimmune disease occurred in around 4 % of patients without having a lymphocyte-depleting agent during the HSCT conditioning regimen, although 9 % of patients suffered from secondary autoimmune disease after using ATG [36].

## Complications compared with the beneficial effects of HSCT in MS

A specific study identifying the toxicity and feasibility of auto-HSCT in 15 patients with SP-MS and RR-MS with a median Expanded Disability Status Scale (EDSS) of 6 (range 4.5–6.5) determined that in two patients neurological deterioration with high fever continued, one patient sustained a transient neural deterioration, three patients exhibited transient engraftment syndrome, one patient had unsuccessful mobilization, and reactivation of cytomegalovirus occurred in one patient. The EDSS improved in three patients assessed at 1-year follow-up after transplantation but remained constant in nine patients and worsened in two patients. According to magnetic resonance imaging (MRI), enhanced T1 lesion disappeared in patients. This study demonstrated the feasibility and acceptable toxicity of using auto-HSCT to reduce the progression of MS. Long-term follow-up after transplantation is vital for the health management of MS patients [37].

A more recent study conducted by Shevchenko et al. [38] set out to determine the long-term effectiveness and safety of auto-HSCT in conjunction with high-dose immunosuppressive therapy (HDIT), along with a decreased intensity BEAM condition regimen, for different types of MS patients. Fassas et al. [39] pioneered BEAM (BCNU (carmustine), cytarabine, etoposide, melphalan) as a conditioning regimen for auto-HSCT, which includes carmustine (bis-chloroethyl nitrosourea), etoposide, cytosine arabinoside, and melphalan. This study involved 99 MS patients (39 male and 60 female, mean age 35 years); 43 of the patients were RR-MS, 35 were SP-MS, 18 were PP-MS, and three were PR-MS with EDSS prior to graft of 3.5. Ninety-eight patients had a neurological improvement or stabilization after 6 months of transplantation [38]. There were no transplanted deaths observed and the cumulative incidence of disease progression was 16.7 % at 8 years post-transplantation. These studies were very favorable because 47 % of the patients improved in their EDSS score (at least 0.5), as compared with the baseline, and 45 % of MS patients were stable at median long-term follow-up for more than 5 years in both the RR-MS and progressive MS groups. Besides, the patient’s quality of life improved in MS by using auto-HSCT with this specific regimen [38]. The consistency observed in these long-term clinical results associated with the quality of life enhancement for these patients reported by Bowen et al. [24] advocates for the safety and efficiency of this treatment approach in MS patients. In addition, a clinical study was conducted by Guimarães et al. [40] to determine the impact of auto-HSCT on health-related quality of life (HRQL) in patients with PP-MS and SP-MS in Brazil [40]. Approximately 79 % of patients enrolled in these trials (27 patients) revealed neurological improvement 1 year after transplantation with significant improvement in the HRQL, indicating that although HSCT involves complicated procedures it impacts positively in MS patients by improving their HRQL. Progression-free survival after treatment with HSCT has been reported in 81 % of SP-MS patients after 3 years and in 67 % of PP-MS patients [40].

On the other hand, researchers in Greece released long-term results from a single-center phase I/II trial of auto-HSCT [41]. According to clinical and MRI data from 35 patients with progressive MS treated with auto-HSCT, researchers demonstrated that HSCT is a therapy for aggressive cases but is not recommended for the vast majority of MS patients with more common RR presentations. Moreover, HSCT has a sustained and impressive effect in suppressing disease activity after a medium follow-up period of 11 (range 2–15) years. At 15 years, disease (progression-free) survival was 44 % for patients with active CNS disease and 10 % for those without. There was an improvement in EDSS scores in 16 patients by 0.5–5.5 for a median of 2 years. However, the EDSS from nine patients did not progress above baseline scores and two patients died from transplant-related complications. MRI data identified a significant reduction in gadolinium-enhancing (Gd^+^) lesions after mobilization, becoming maximal post-transplantation [41]. This study is consistent with Saccardi et al. [42] on 178 patients from 45 centers.

Several studies have shown the effectiveness of using HSCT for young patients with highly aggressive, malignant forms of MS. This type of MS is rare and characterized by abnormally localized autoimmune processes in the brain stem or upper cervical cord and/or intense inflammation resulting in rapid development of significant disability or death in the early stages of the disease course. Marburg variant MS is another demyelinating disease with an equally ominous prognosis with malignant MS, although it may differ histologically [43, 44]. Since 2011, researchers in the United Kingdom have evaluated the effect of using pulsed cyclophosphamide and nonmyeloablative auto-HSCT in the case of a severely disabled 21-year-old female patient presenting with malignant MS. This treatment led to improvement in the patient, who suffered from a disturbance of sensorimotor function in all four limbs. The results of her neuroimaging showed demyelination with enhancement, cerebrospinal fluid (CSF) was positive to oligoclonal bands and demonstrated 47 lymphocytes, and serum was negative for aquaporin4 (Aq4) antibodies. Although the patient experienced several treatments, such as alemtuzumab with pulsed intravenous methylprednisolone (IVMP) and plasma exchange, she continued to deteriorate. The patient underwent auto-HSCT after 2 months of treatment with cyclophosphamide. The patient’s EDSS score was 8.5 at the time of transplantation but 1 year after treatment the score decreased to 6.5, and she was able to walk. According to this case report, using cyclophosphamide prior to auto-HSCT is crucial for clinical improvement, suppression of relapses, and stabilization of the lesion burden, especially in highly active RR-MS patients [45]. This study is consistent with the study of Faguis et al. [46] on nine patients with malignant RR-MS who underwent auto-HSCT with BEAM condition followed by cyclophosphamide, resulting in one relapse in 280 patient-months following HSCT. All patients had their disability improve or stabilize, and most of the patients showed no enhanced lesions during follow-up. Furthermore, Kimiskidis et al. [44] reported an improvement and long-lasting clinical and radiological response in a case with malignant MS who was treated with high-dose chemotherapy plus ATG followed by auto-HSCT.

Results from several studies recommend nonmyeloablative HSCT to treat SP-MS and RR-MS mainly due to the fact that HSCT has immunosuppressive and immunomodulatory effects, evident from a more diverse T-cell clonal population in post-HSCT patients [47]. This occurs due to the ability of HSCT to modulate autoimmunity without the requirement to eradicate the full patient’s hematopoietic cells (myeloablation regimen). In 2015, a study by Burt et al. [48] focused on improving neurological disabilities and other clinical results of RR-MS patients using nonmyeloablative HSCT. This study included 191 MS patients; 123 patients had a RR course of the disease and 28 had SP-MS, with mean age of 36 years. Patients were treated at a single US institution between 2003 and 2014, and the researchers followed-up with patients for 5 years. At year 4 post-transplantation, 64 % of patients demonstrated improvement in the EDSS score from a pretransplant median of 4.0 to 2.5, neurological rating scale scores increased from a pretransplant median of 74 to 87.5 in 34 patients, and the MS functional composite scores were 0.45 (0.04–0.60) (*p* = 0.02). The brain T2 lesion volume reduced significantly from a pretransplant median of 8.57 cm^3^ to 5.74 cm^3^ (*p* < 0.001) at the last post-transplant assessment of MRI scans in 128 patients with a mean follow-up of 27 months. In this study, the quality-of-life short form based upon the 36 questionnaire score was improved significantly in 132 patients from a pretransplant median of 46 to 64 at year 2 post-transplant as compared with the previous results of the AFFIRM (The Natalizumab Safety and Efficacy in Relapsing Remitting Multiple Sclerosis) and SENTINEL (The Safety and Efficacy of Natalizumab in Combination with Interferon Beta-1a in Patients with Relapsing Remitting Multiple Sclerosis) trials. In addition, there were no early or late infectious cases of fungal, *Pneumocystis jirovecii*, JC virus, Epstein–Barr virus, or cytomegalovirus, and there was no treatment-related mortality [48]. Altogether, using nonmyeloablative HSCT was crucial in improving neurological disabilities in RR-MS patients.

A major pathological complication of auto-HSCT may be the effects reported on brain volume. Saiz et al. [49] focused on monitoring the evolution of inflammatory disease activity by suppressing the relapses and Gd^+^ MRI lesions post-auto-HSCT. Four out of five patients had a constant or improved EDSS after 3 and 12 months post-transplantation, but the fifth patient suffered deterioration in their condition during the treatment. For all of the patients post-auto-HSCT, there was no enhancement of T1 lesions and no enlargement of or new T2 lesions (median: 11.8 % appearing). In addition, the corpus callosum area decreased in all patients at 1-year follow-up (median declines: 12.4 %) and for two patients there was no progress at 2 years post-HSCT. These results suggest a positive impact of auto-HSCT on active inflammation that corresponds with the clinical stabilization of the five patients at 1 year post-HSCT. Although the five patients showed improvements in other MRI variables, the atrophy of the corpus callosum increased. The relationship between the development of brain atrophy and inflammatory activity is uncertain. This study indicates the effectiveness of using auto-HSCT in arresting the inflammatory activity; however, the pathological process responsible for brain atrophy was not reversed [49].

Rapid loss of brain volume has been measured a few months after treatment [50]. Auto-HSCT has seemingly detrimental effects on the integrity of the brain tissue that leads to rapid loss of about 1.92 % of brain volume. A study of SP-MS patients showed that brain atrophy after auto-HSCT is not constant but declines for the first 2 years after treatment. The reduction in brain volume may be a result of the significant inflammation seen before stem cell transplantation is performed. The pathological evidence for this is through the large number of transected axons seen in MS lesions, marked axonal injury, cortical demyelination, and diffuse inflammation of the brain observed after histopathological analysis [50]. Histopathological studies have also been carried out on brain tissue obtained at autopsy from deceased patients who had been treated with auto-HSCT, and they indicate ongoing active demyelination. Metz et al. [51] interrogated brain tissue samples from five patients with chronic lesions where they investigated 53 individual white matter lesions through immunohistochemical and routine staining techniques. They were able to characterize damaged axons, activated macrophages/microglial cells, inflammatory infiltrates, and demyelinating activity in these lesions. Limited numbers of T cells, which were dominated by CD8^+^ cytotoxic T cells in the inflammatory infiltrate, could be observed within the lesions while plasma cells and B cells were completely absent. Macrophages/microglial cells were found on the injured tissue and high numbers of damaged axons were present in the active lesion areas. The study concluded that axonal degeneration and demyelination remain a constant feature even after auto-HSCT, despite effective immunosuppression associated with transplantation [51]. Their data were supported by other clinical studies, which indicate that there is continued clinical disease progression in MS patients with high EDSS despite their special auto-HSCT therapy [52]. The clinical efficacy of intense immunosuppression with auto-HSCT does not appear to avoid further progression in MS patients with high EDSS score (>6.0) [51]. Thus, future HSCT trials should consider inclusion criteria to be an early stage of disease course with active relapses of MS, as well as combinatorial new therapeutic strategies that may prevent ongoing neurodegeneration and demyelination in progressive MS [51].

A more recent study undertaken by Mancardi et al. [53] in Italy evaluated the effect of a highly intense conditioning regimen followed by auto-HSCT compared with the immunosuppressive therapy mitoxantrone (MTX, Novantrone; EMD Serono, Rockland, MA USA) on the disease activity of 21 MS patients. This controlled, randomized, multicenter phase II trial of auto-HSCT led to a significant reduction in T2-weighted lesions, Gd^+^ areas, and annualized relapse rates (ARR) as compared with MTX. Nine patients (four SP-MS with relapses, three SP-MS, two RR-MS) were assigned to the auto-HSCT group and 12 patients ended up in the MTX-treatment group (five RR-MS, three SP-MS, three SP-MS with relapses, one RP-MS). The first group was given 4 g/m^2^ cyclophosphamide and 5 μg/kg body weight filgrastim, along with a high-dose chemotherapy conditioning regimen, BEAM. Patients treated with MTX received a 20 mg MTX dose intravenously and 1 g methylprednisolone diluted in 250 ml 0.9 % saline every month for 6 months. The researchers found that 79 % of patients who experienced auto-HSCT had fewer new T2 lesions on MRI scans as compared with the MTX-treated patients by using an intention-to-treat analysis. However, due to patient dropout and technical problems, only 17 of the 21 patients had MRI scans. Furthermore, there was a complete suppression of active inflammatory lesions, as demonstrated by the absence of new Gd^+^ lesions in the auto-HSCT group during a 4-year follow-up, although 56 % of MTX-treated patients had inflammatory activity. Regarding the effect of ARR over 4 years, ARR reduced in the auto-HSCT patients (ARR = 0.19) as compared with MTX patients (ARR = 0.6). Nevertheless, only 48 % of MTX patients demonstrated progression while 57 % of auto-HSCT patients had progressed at the end of follow-up. There were no observable differences in EDSS scores between the two groups. This study demonstrated that for patients with severe progressive MS, treatment with auto-HSCT is superior to treatment with MTX. The effect is related to the intensity of the conditioning regimen, which was used to reset the immune system in MS patients before performing HSCT, compared with the Nash et al. [54] and Burt et al. [48] trials that were reported earlier in 2015. Although this randomized and controlled study yielded promising results, the sample size was small. In addition, the clinical results of the study by Mancardi et al. [53] were lackluster in comparison with other studies in auto-HSCT, especially when compared with the reduction in EDSS among MS patients who enrolled in the Burt et al. [48] study in 2015 as well as the information on quality of life and brain atrophy, not investigated in this study.

## Criteria to be considered in HSCT in MS patients

There are several criteria that may play a crucial role in using HSCT as a therapeutic option in the management of MS, including proposed international multicenter, randomized clinical trials of HSCT compared with the best standard of care treatment, MS patient selection in studies, and long-term follow-up studies of patients from international registries [29]. Although recent studies provide a significant improvement in various types of MS patients’ lives, several vital limitations have been reported [38, 48]. Firstly, the inferences deducted from HSCT effects cannot be made because most studies were observational for the treated cohorts without appropriate control groups. Secondly, there has not been enough information about disease activity before the disease course and its treatment (and there has been no long-term follow-up available for certain patients). In addition, the studies were mostly performed at a single institution, which may implicate bias.

## Conclusion

HSCT is a plausible treatment paradigm for MS patients. However, auto-HSCT is considered to be a sledgehammer approach for treating MS patients, one that will be astoundingly effective when used on appropriately selected patients. The reasons for this promising therapy’s success are its lower toxicity and its ability to replace the immune system. The future of HSCT trials should discover novel therapeutic strategies that prevent ongoing neurodegeneration and demyelination in progressive MS. The trial designs should consider the reproducibility of HSCs with sufficient yield and purity, select MS patients with active inflammatory disease, and use appropriate conditioning agents.

## Abbreviations

5-FU: 5-Fluorouracil
ALDH: Aldehyde dehydrogenase
ARR: Annualized relapse rates
ATG: Anti-thymocyte globulin
auto-HSCT: Autologous hematopoietic stem cell transplantation
BBB: Blood–brain barrier
BM: Bone marrow
CFA: Complete Freund’s adjuvant
CFU-S: Colony-forming spleen assays
CNS: Central nervous system
CSF: Cerebrospinal fluid
EAE: Experimental autoimmune encephalomyelitis
EDSS: Expanded Disability Status Scale
FACS: Fluorescence-activated cell sorting
G-CSF: Granulocyte colony-stimulating factor
Gd^+^: Gadolinium-enhancing
GVHD: Graft versus host disease
HDIT: High-dose immunosuppressive therapy
HRQL: Health-related quality of life
HSC: Hematopoietic stem cell
HSCT: Hematopoietic stem cell transplantation
IVMP: Intravenous methylprednisolone
LSK: Lin^–^, Sca-1^+^, and c-Kit^+^
LT: Long term
MACS: Magnetic-activated separation
MBP: Myelin basic protein
MOG: Myelin oligodendrocyte glycoprotein
MRI: Magnetic resonance imaging
MS: Multiple sclerosis
MTX: Mitoxantrone
PBCS: Peripheral blood stem cells
PLP: Proteolipid protein
PP: Primary progressive
PR: Progressive relapsing
RR: Relapsing remitting
SP: Secondary progressive
ST: Short term
TBI: Total body irradiation

## References

[1] Villar LM, Espino M, Cavanillas ML, Roldan E, Urcelay E, de la Concha EG (2010). Immunological mechanisms that associate with oligoclonal IgM band synthesis in multiple sclerosis. Clin Immunol.

[2] Vosoughi R, Freedman MS (2010). Therapy of MS. Clin Neurol Neurosurg.

[3] Fassas A, Mancardi GL (2008). Autologous hemopoietic stem cell transplantation for multiple sclerosis: is it worthwhile?. Autoimmunity.

[4] Weinshenker BG, Bass B, Rice GP, Noseworthy J, Carriere W, Baskerville J (1989). The natural history of multiple sclerosis: a geographically based study. I. Clinical course and disability. Brain.

[5] Compston A, Coles A (2002). Multiple sclerosis. Lancet.

[6] Fassas A, Nash R (2004). Multiple sclerosis. Best Pract Res Clin Haematol.

[7] Scolding N (2011). Adult stem cells and multiple sclerosis. Cell Prolif.

[8] Van Wijmeersch B, Sprangers B, Dubois B, Waer M, Billiau AD (2008). Autologous and allogeneic hematopoietic stem cell transplantation for multiple sclerosis: perspective on mechanisms of action. J Neuroimmunol.

[9] Burt RK, Burns W, Hess A (1995). Bone marrow transplantation for multiple sclerosis. Bone Marrow Transplant.

[10] Fassas A, Anagnostopoulos A, Kazis A, Kapinas A, Sakellari I, Kimiskidis V (1997). Peripheral blood stem cell transplantation in the treatment of progressive multiple sclerosis: first results of a pilot study. Bone Marrow Transplant.

[11] Filip S, Mokrý J, Vávrová J, Cízková D, Sinkorová Z, Tosnerová V (2009). Homing of lin–/CD117+ hematopoietic stem cells. Transfus Apher Sci.

[12] Challen GA, Boles N, Lin KK, Goodell MA (2009). Mouse hematopoietic stem cell identification and analysis. Cytometry A.

[13] Wognum AW, Eaves AC, Thomas TE (2003). Identification and isolation of hematopoietic stem cells. Arch Med Res.

[14] Juopperi TA, Schuler W, Yuan X, Collector MI, Dang CV, Sharkis SJ (2007). Isolation of bone marrow-derived stem cells using density-gradient separation. Exp Hematol.

[15] Maetzig T, Brugman MH, Bartels S, Heinz N, Kustikova OS, Modlich U (2011). Polyclonal fluctuation of lentiviral vector-transduced and expanded murine hematopoietic stem cells. Blood.

[16] Tsiftsoglou AS, Bonovolias ID, Tsiftsoglou SA (2009). Multilevel targeting of hematopoietic stem cell self-renewal, differentiation and apoptosis for leukemia therapy. Pharmacol Ther.

[17] Pearce DJ, Bonnet D (2007). The combined use of Hoechst efflux ability and aldehyde dehydrogenase activity to identify murine and human hematopoietic stem cells. Exp Hematol.

[18] Bettelli E (2007). Building different mouse models for human MS. Ann N Y Acad Sci.

[19] Chan J, Ban E, Chun K, Wang B, Backstrom B, Bernarcd C (2008). Transplantation of bone marrow transduced to express self-antigen establishes deletional tolerance and permanently remits autoimmune disease. J Immunol.

[20] Alderuccio F, Nasa Z, Chung J, Ko HJ, Chan J, Toh BH (2011). Hematopoietic stem cell gene therapy as a treatment for autoimmune diseases. Mol Pharm.

[21] Burt RK, Testori A, Craig R, Cohen B, Suffit R, Barr W (2008). Hematopoietic stem cell transplantation for autoimmune diseases: what have we learned?. J Autoimmun.

[22] van Gelder M, van Bekkum DW (1996). Effective treatment of relapsing experimental autoimmune encephalomyelitis with pseudoautologous bone marrow transplantation. Bone Marrow Transplant.

[23] van Bekkum D (2004). Stem cell transplantation for autoimmune disorders. Preclinical experiments. Best Pract Res Clin Haematol.

[24] Bowen J, Kraft GH, Wundes A, Guan Q, Maravilla KR, Gooley TA (2012). Autologous hematopoietic cell transplantation following high-dose immunosuppressive therapy for advanced multiple sclerosis: long-term results. Bone Marrow Transplant.

[25] Burt RK, Cohen B, Rose J, Petersen F, Oyama Y, Stefoski D (2005). Hematopoietic stem cell transplantation for multiple sclerosis. Arch Neurol.

[26] Farge D, Labopin M, Tyndall A, Fassas A, Mancardi GL, Van Laar J (2010). Autologous hematopoietic stem cell transplantation for autoimmune diseases: an observational study on 12 years’ experience from the European Group for Blood and Marrow Transplantation Working Party on Autoimmune Diseases. Haematologica.

[27] Pasquini MC, Voltarelli J, Atkins HL, Hamerschlak N, Zhong X, Ahn KW (2012). Transplantation for autoimmune diseases in North and South America: a report of the Center for International Blood and Marrow Transplant Research. Biol Blood Marrow Transplant.

[28] Openshaw H, Stuve O, Antel JP, Nash R, Lund BT, Weiner LP (2000). Multiple sclerosis flares associated with recombinant granulocyte colony-stimulating factor. Neurology.

[29] Atkins HL, Freedman MS (2013). Hematopoietic stem cell therapy for multiple sclerosis: top 10 lessons learned. Neurotherapeutics.

[30] Gress RE, Emerson SG, Drobyski WR (2010). Immune reconstitution: how it should work, what’s broken, and why it matters. Biol Blood Marrow Transplant.

[31] Sureda A, Bader P, Cesaro S, Dreger P, Duarte RF, Dufour C (2015). Indications for allo- and auto-SCT for haematological diseases, solid tumours and immune disorders: current practice in Europe, 2015. Bone Marrow Transplant.

[32] Muraro PA, Douek DC, Packer A, Chung K, Guenaga FJ, Cassiani-Ingoni R (2005). Thymic output generates a new and diverse TCR repertoire after autologous stem cell transplantation in multiple sclerosis patients. J Exp Med.

[33] Gosselin D, Rivest S (2011). Immune mechanisms underlying the beneficial effects of autologous hematopoietic stem cell transplantation in multiple sclerosis. Neurotherapeutics.

[34] Loh Y, Oyama Y, Statkute L, Traynor A, Satkus J, Quigley K (2007). Autologous hematopoietic stem cell transplantation in systemic lupus erythematosus patients with cardiac dysfunction: feasibility and reversibility of ventricular and valvular dysfunction with transplant-induced remission. Bone Marrow Transplant.

[35] Daikeler T, Labopin M, Di Gioia M, Abinun M, Alexander T, Miniati I (2011). Secondary autoimmune diseases occurring after HSCT for an autoimmune disease: a retrospective study of the EBMT Autoimmune Disease Working Party. Blood.

[36] Cuker A, Coles AJ, Sullivan H, Fox E, Goldberg M, Oyuela P (2011). A distinctive form of immune thrombocytopenia in a phase 2 study of alemtuzumab for the treatment of relapsing-remitting multiple sclerosis. Blood.

[37] Carreras E, Saiz A, Marin P, Martinez C, Rovira M, Villamor N (2003). CD34+ selected autologous peripheral blood stem cell transplantation for multiple sclerosis: report of toxicity and treatment results at one year of follow-up in 15 patients. Haematologica.

[38] Shevchenko JL, Kuznetsov AN, Ionova TI, Melnichenko VY, Fedorenko DA, Kurbatova KA (2015). Long-term outcomes of autologous hematopoietic stem cell transplantation with reduced-intensity conditioning in multiple sclerosis: physician’s and patient’s perspectives. Ann Hematol.

[39] Fassas A, Anagnostopoulos A, Kazis A, Kapinas K, Sakellari I, Kimiskidis V (2000). Autologous stem cell transplantation in progressive multiple sclerosis—an interim analysis of efficacy. J Clin Immunol.

[40] Guimarães FA, Oliveira-Cardoso EA, Mastropietro AP, Voltarelli JC, Santos MA (2010). Impact of autologous hematopoetic stem cell transplantation on the quality of life of patients with multiple sclerosis. Arg Neuropsiquiatr.

[41] Fassas A, Kimiskidis VK, Sakellari I, Kapinas K, Anagnostopoulos A, Tsimourtou V (2011). Long-term results of stem cell transplantation for MS: a single-center experience. Neurology.

[42] Saccardi R, Kozak T, Bocelli-Tyndall C, Fassas A, Kazis A, Havrdova E (2006). Autologous stem cell transplantation for progressive multiple sclerosis: update of the European Group for Blood and Marrow Transplantation autoimmune diseases working party database. Mult Scler.

[43] Atkins HL, Muraro PA, van Laar JM, Pavletic SZ (2012). Autologous hematopoietic stem cell transplantation for autoimmune disease—is it now ready for prime time?. Biol Blood Marrow Transplant.

[44] Kimiskidis V, Sakellari I, Tsimourtou V, Kapina V, Papagiannopoulos S, Kazis D (2008). Autologous stem-cell transplantation in malignant multiple sclerosis: a case with a favorable long-term outcome. Mult Scler.

[45] Alix JJ, Blackburn DJ, Sokhi D, Craven I, Sharrack B, Snowden JA (2013). Autologous hematopoietic stem cell transplantation following pulsed cyclophosphamide in a severely disabled patient with malignant multiple sclerosis. J Neurol.

[46] Fagius J, Lundgren J, Oberg G (2009). Early highly aggressive MS successfully treated by hematopoietic stem cell transplantation. Mult Scler.

[47] Holloman J, Ho CC, Hukki A, Huntley JL, Gallicano GI (2013). The development of hematopoietic and mesenchymal stem cell transplantation as an effective treatment for multiple sclerosis. Am J Stem Cells.

[48] Burt RK, Balabanov R, Han X, Sharrack B, Morgan A, Quigley K (2015). Association of nonmyeloablative hematopoietic stem cell transplantation with neurological disability in patients with relapsing-remitting multiple sclerosis. JAMA.

[49] Saiz A, Carreras E, Berenguer J, Yague J, Martinez C, Marin P (2001). MRI and CSF oligoclonal bands after autologous hematopoietic stem cell transplantation in MS. Neurology.

[50] Burt RK, Cohen BA, Russell E, Spero K, Joshi A, Oyama Y (2003). Hematopoietic stem cell transplantation for progressive multiple sclerosis: failure of a total body irradiation-based conditioning regimen to prevent disease progression in patients with high disability scores. Blood.

[51] Metz I, Lucchinetti CF, Openshaw H, Garcia-Merino A, Lassmann H, Freedman MS (2007). Autologous haematopoietic stem cell transplantation fails to stop demyelination and neurodegeneration in multiple sclerosis. Brain.

[52] Sainaghi PP, Collimedaglia L, Alciato F, Molinari R, Sola D, Ranza E (2013). Growth arrest specific gene 6 protein concentration in cerebrospinal fluid correlates with relapse severity in multiple sclerosis. Mediators Inflamm.

[53] Mancardi GL, Sormani MP, Gualandi F, Saiz A, Carreras E, Merelli E (2015). Autologous hematopoietic stem cell transplantation in multiple sclerosis: a phase II trial. Neurology.

[54] Nash RA, Hutton GJ, Racke MK, Popat U, Devine SM, Griffith LM (2015). High-dose immunosuppressive therapy and autologous hematopoietic cell transplantation for relapsing-remitting multiple sclerosis (HALT-MS): a 3-year interim report. JAMA Neurol.

